# 

**Published:** 1947

**Authors:** 


### CONTENTS.

| | Page |
|---|---|
| Diagnosis of Disease in Infancy. By A. V. Neale, M.D., F.R.C.P. | 93 |
| Psychosomatic Dermatoses. By W. J. O'Donovan, M.D., O.B.E. | 98 |
| Poliomyelitis in Bristol, 1947. By James Macrae, M.D., F.R.F.P.S., D.P.H. | 101 |
| House of John Wright | 106 |
| Reviews of Books | 108 |
| The Medical Library of the University of Bristol | 111 |
| Publications Received | 113 |
| Local Medical Notes | 114 |

